# Supplementary figures and images for: Evaluation of shotgun metagenomics as a diagnostic tool for infectious gastroenteritis
Source: PLoS One. 2025 Sep 2;20(9):e0331288. doi: 10.1371/journal.pone.0331288 (PMC12404398; doi:10.1371/journal.pone.0331288)

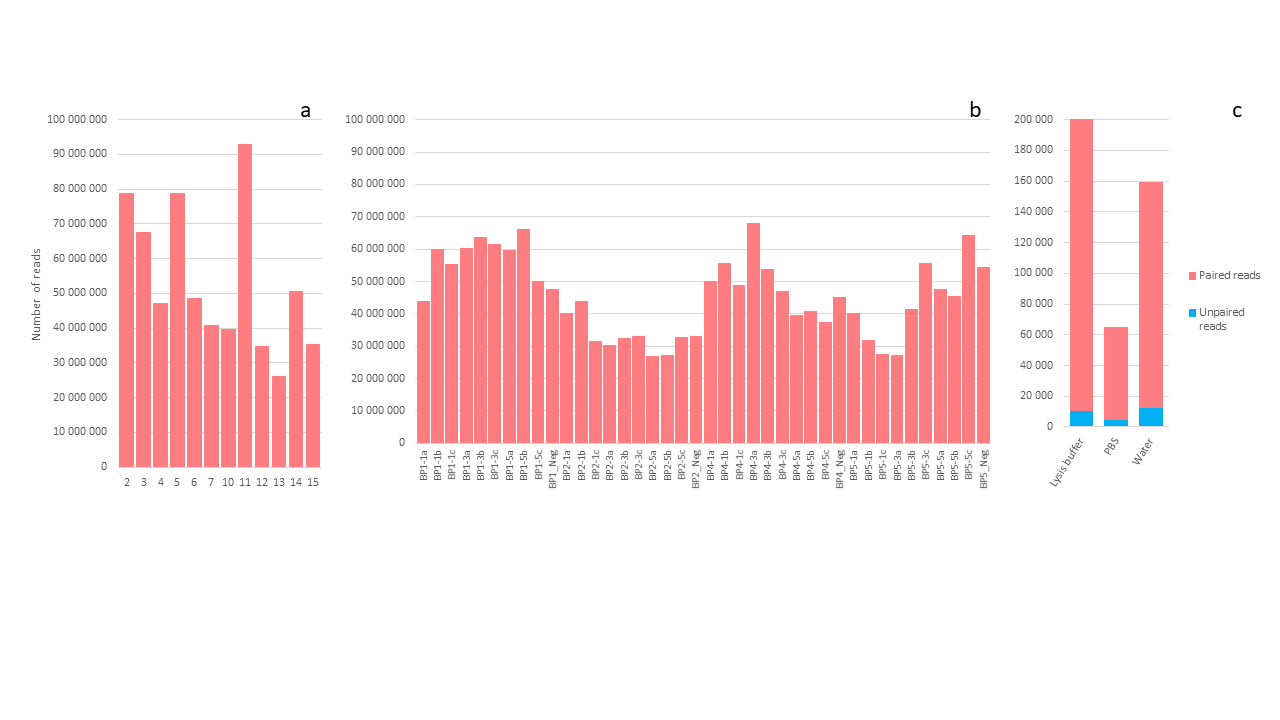

Supplement: S1 Fig — Read statistics are shown after quality control filtering for clinical samples, spiked samples, and negative controls. (TIF) [file pone.0331288.s001.tif]
