# Supplementary material for: Evaluation of shotgun metagenomics as a diagnostic tool for infectious gastroenteritis
Source: PLoS One. 2025 Sep 2;20(9):e0331288. doi: 10.1371/journal.pone.0331288 (PMC12404398; doi:10.1371/journal.pone.0331288)
Supplement: S6 Table — (DOCX) [file pone.0331288.s007.docx]

Supplementary Table 6: Average number of reads from clinical and spiked faecal samples mapping to *Giardia* spp. reference genomes.

| Sample | *G. intestinalis* (GCA_000002435.2) | *G. lamblia*  (GCA_000182665.1) | *G. muris*  (GCA_006247105.1) |
| --- | --- | --- | --- |
| 10 | 367 | 410 | 22 |
| BP1 | 68 | 6 | 3 |
| BP2 | 16 | 3 | 40 |
| BP4 | 40 | 9 | 0 |
| BP5 | 14 | 2 | 1 |
